# Epithelial-Mesenchymal Transition Induces Endoplasmic-Reticulum-Stress Response in Human Colorectal Tumor Cells

**DOI:** 10.1371/journal.pone.0087386

**Published:** 2014-01-31

**Authors:** Evelyn Zeindl-Eberhart, Lydia Brandl, Sibylle Liebmann, Steffen Ormanns, Silvio K. Scheel, Thomas Brabletz, Thomas Kirchner, Andreas Jung

**Affiliations:** 1 Institute of Pathology, University of Munich, Munich, Germany; 2 Department of Visceral Surgery, University of Freiburg, Freiburg, Germany; University of Alabama at Birmingham, United States of America

## Abstract

Tumor cells are stressed by unfavorable environmental conditions like hypoxia or starvation. Driven by the resulting cellular stress tumor cells undergo epithelial-mesenchymal transition. Additionally, cellular stress is accompanied by endoplasmic reticulum-stress which induces an unfolded protein response. It is unknown if epithelial-mesenchymal transition and endoplasmic reticulum-stress are occurring as independent parallel events or if an interrelationship exists between both of them. Here, we show that in colorectal cancer cells endoplasmic reticulum-stress depends on the induction of ZEB-1, which is a main factor of epithelial-mesenchymal transition. In the absence of ZEB-1 colorectal cancer cells cannot mount endoplasmic reticulum-stress as a reaction on cellular stress situations like hypoxia or starvation. Thus, our data suggest that there is a hierarchy in the development of cellular stress which starts with the presence of environmental stress that induces epithelial-mesenchymal transition which allows finally endoplasmic reticulum-stress. This finding highlights the central role of epithelial-mesenchymal transition during the process of tumorigenesis as epithelial-mesenchymal transition is also associated with chemoresistance and cancer stemness. Consequently, endoplasmic reticulum-stress might be a well suited target for chemotherapy of colorectal cancers.

## Introduction

Colorectal carcinoma (CRC) belongs to the most prevalent cancer types in the Western world. Many CRCs are characterized by infiltrative growth which is associated with epithelial-mesenchymal transition (EMT). EMT is frequently seen at the invasive front of CRCs and is accompanied by an increased expression of mesenchymal marker genes like vimentin [1], [2]. Prerequisites for EMT are changes in the epithelial phenotype of tumor cells, like loss of epithelial polarity and the zonula adhaerentes as well as a gain of mobility [1], [2]. Loss of zonula adhaerentes is mediated by downregulation of E-cadherin, a cell adhesion molecule localized at the cell membrane of epithelial cells. Transcription of E-cadherin during EMT is regulated by different transcriptional repressors like zinc finger E-box binding protein (ZEB1), which represents the most important factor for EMT induction in CRCs [3]–[5]. ZEB1 was found immunhistochemically at the front of invasion in colorectal carcinomas but not in central tumor areas supporting the importance of ZEB1 for EMT [6].

In the zonula adhaerentes E-cadherin is linked to β-catenin which is also a component of the canonical Wnt signaling pathway that is frequently deregulated in CRCs [7]. Loss of E-cadherin causes the release of β-catenin from the zonula adhaerentes. As a consequence β-catenin might enter the nucleus where it associates with members of the T-cell factor (TCF)/lymphocyte enhancing factor-1 (LEF1) family. Such dimers are the central element of a multiprotein complex that promotes transcription of β-catenin target genes which mediate migration, invasion, EMT and other hallmarks of cancer [7], [8].

Due to their high proliferation and metabolism on the one hand and poor vascularisation of tumors on the other hand, tumor cells suffer from pathophysiological environmental conditions like glucose starvation, hypoxia, acidosis, low pH value and others. In particular hypoxia is an important driver of tumorigenesis. It is mainly regulated by the transcriptional activator hypoxia-inducible factor-1 (HIF1), a heterodimer composed of the subunits HIF1α and HIF1β. During hypoxia HIF1α degradation is blocked and HIF1α accumulates subsequently also in the nucleus. Here, HIF1α dimerizes with HIF1β and the resulting HIF1 complex binds to specific hypoxia responsive elements in the promoter/enhancer region of its target genes, thereby activating the angiogenic switch. This leads to reperfusion and thus to an increase in oxygen availability and thus a release of hypoxia [9], [10].

Hypoxia also affects the function of the endoplasmic reticulum (ER) causing an accumulation of unfolded, misfolded or aggregated proteins within the ER lumen. This leads to an imbalance between the cellular demand for ER-function and ER-capacity resulting in ER-stress [11]. To reduce ER-stress and to ensure cell survival, a highly conserved intracellular signalling cascade, the unfolded protein response (UPR), is initiated. UPR is accompanied by the activation of different ER-resident transmembrane stress sensors, like the activating transcription factor-6 (ATF6) [12]. In the absence of cellular stress the function of the stress sensors is blocked by the molecular chaperone 78 kD glucose-regulated protein (GRP78), which is located in the ER lumen and the key protein initiating ER-stress response [12]. Under cellular stress misfolded proteins accumulate and bind to GRP78 resulting in the release and activation of the stress sensors [12], [13]. When GRP78 dissociates from ATF6 it translocates into the Golgi complex, where it is cleaved into a transcriptionally active 50 kD fragment that subsequently enters the nucleus. Here, it binds to ER-stress response elements of several genes like GRP78 and activate their expression. Thus, both enhanced GRP78 amounts and the presence of the 50 kD ATF6 cleavage product are indicators of an ER-stress response [14].

It is noteworthy that EMT and hypoxia have several characteristics in common, like induction of invasiveness, expression of mesenchymal marker genes as vimentin [1], [2] as well as the loss of E-cadherin [1], [2], [15], [16]. Interestingly, at the invasive front of CRCs, tumor cells displaying EMT [1], [2] are characterized by nuclear β-catenin as well as nuclear HIF1α [2], [17]. Thus, ER-stress on the one hand, and EMT on the other hand occur in parallel in tumor cells. It is currently unknown if there is an interrelation between EMT and ER-stress which might connect the two main drivers of colorectal carcinogenesis namely Wnt/β-catenin signalling and hypoxia. Here, we show that EMT induces an ER-stress response which assists in and raises tumor cell survival.

## Materials and Methods

According to the guidelines of our University, immunohistochemical staining of archived tissue samples may be performed provided that anonymity is granted. Therefore, approval of this study was waived by the ethical committee of the University of Munich.

### Cell Culture

The human cultured CRC cell lines SW480 and HCT116 (American Type Culture Collection, Manasas, VA, USA) are both characterized by aberrantly activated Wnt/β-catenin signaling [18]. The SW480 derivatives SW480-shZEB1 (with stable short hairpin RNA (shRNA)-mediated knockdown of ZEB1, further designated as SW480-shZEB1) and SW480-shGFP (stably express ZEB1 and shGFP (green fluorescent protein (GFP), further designated as SW480-controls) were used kindly provided by Prof. Th. Brabletz, Freiburg, Germany [3]. All cell lines were cultured in Dulbecco's modified essential medium (DMEM)/Ham's F12 (1∶1) medium with stable L-glutamine (365.3 mg/l; Biochrom, Berlin, Germany) and 10% (v/v) fetal bovine serum (FBS) at 37°C in a humified 5% CO_2_ atmosphere. The identity of all cell lines was authenticated at the DSMZ (Deutsche Sammlung für Mikroorganismen und Zellkulturen GmbH, Braunschweig, Germany) in September, 2011 and then rechecked semiannually in the laboratory using the same marker set as DSMZ.

### EMT induction

Two methods were used for the induction of EMT. 1. growth density [19]. Therefore, for each experiment tumor cells were seeded at high density (∼7×10^4^/cm^2^) on a petri dish (10 cm diameter) and harvested after 72–96 h at confluence, when they displayed an epithelial phenotype [19]. Additionally cells were seeded at low density (∼7×10^3^/cm^2^) on three petri dishes (10 cm diameter) and harvested after 48–52 h when they exhibited a mesenchymal phenotype. Four experiments were conducted per cell line each consisting of one low density and two confluent growing samples. 2. serum starvation [20]. Therefore, tumor cells were seeded on petri dishes (3 cm diameter) and grown to confluence in normal culture medium. Then cells were either grown for additional 12 h in normal medium, or serum starved for 6 h in serum-free medium which was replaced by FBS containing medium. Cells were harvested after 6 h. Serum starvation experiments were conducted three times.

### Induction of hypoxia and reoxygenation

For the induction of hypoxia confluent growing SW480 and HCT116 cells seeded on petri dishes (3 cm diameter) were treated with 100 µM of cobalt chloride (CoCl_2_) (Sigma-Aldrich, Taufkirchen, Germany), in serum free medium for 1–9 h [21]. For reoxygenation confluent growing SW480-shZEB1 cells and SW480-controls seeded on petri dishes (3 cm diameter) were treated with 100 µM CoCl_2_ in serum free medium for 6 h. After washing cells were incubated in normal medium without CoCl_2_ and harvested after 0.5 h, 1.0 h and from then on in 3.0 h intervals up to 24 h. Both experiments were done three times.

### Preparation of protein samples

For the determination of EMT-associated proteins by two-dimensional electrophoresis (2-DE) whole cellular proteins were extracted from confluent and sparsely growing SW480 and HCT116 cells [22]. Protein samples were mixed with urea (9M), DTT (70 mM) (both BioRad, Munich, Germany) and Ampholytes (2%, Servalytes pH 5–7, Serva, Heidelberg, Germany), cleared by centrifugation (14,000 rpm/10 min/4°C) and frozen in liquid nitrogen. For standard polyacrylamide gel electrophoresis (PAGE) proteins were extracted with triple-detergent lysis buffer [23] supplemented with a protease inhibitor cocktail [22], cleared by centrifugation and finally frozen in liquid nitrogen.

### Protein detection

Changes in EMT-associated protein composition were determined by comparative proteome analysis employing 2-DE and matrix–assisted laser-desorption/ionization time-of-flight mass spectrometry (MALDI-TOF MS). Protein samples were separated by high-resolution 2-DE using the Iso-Dalt-equipment (Hoefer Scientific Instruments, Kehl, Germany). 75 µg (analytical scale) or 400 µg (preparative scale) proteins were separated in the first dimension by isoelectric focusing [24]. Separation in the second dimension was done using polyacrylamide gradient gels (10%–16%) followed by silver- or Coomassie Brilliant Blue staining (CBB; R250, Merck, Germany) [24]. 2-DE gels resulting from either epithelially (confluent) or mesenchymally (sparsely) grown SW480- or HCT116 cells respectively were compared per cell line pair wise. Protein spots showing differences in intensities were verified by cross comparison of all 2-DE gels within one series [25]. Gel comparisons were done using the image analysis software program TOPSPOT (http://www.mpiib-berlin.mpg.de/2D-PAGE/). An entire 2-DE gel is shown in Figure S1A.

### Protein identification

For protein identification spots of interest were excised from CBB-stained gels and subjected to tryptic in-gel digestion. Peptide masses were determined by the protein analysis unit at the Adolf-Butenandt-Institute of the university of Munich using a MALDI-TOF mass spectrometer (Voyager-DESTR, Applied Biosystems, Darmstadt, Germany) and identified by peptide mass fingerprinting (PMF) searching National Centre for Biotechnology Information and SwissProt databases using the MS-Fit (http://prospector.ucsf.edu/) and PeptIdent software (http://au.expasy.org/tools/peptident.html), respectively.

Sodium dodecyl-sulfate (SDS)-PAGE and immunostaining were used for all subsequent studies [25]. Protein samples were enriched with 2XSDS sample buffer (final concentration: 2% (w/v) SDS, 70 mM DTT, 10% (v/v) Glycerol), resolved using Mini Protean II™ devices (BioRad) using 10% (w/v) polyacrylamide gels, transferred onto hydrophobic polyvinylidene difluoride (PVDF) membranes (pore diameter 0.2 µm, BioRad) under semi dry conditions (1 h at 1 mA/cm^2^), blocked for 1 h in phosphate buffered saline (PBS)/0.1% (v/v) Tween20, 5% (w/v) non-fat dry milk powder (BioRad), incubated overnight (4°C) in the presence of the first antibody and treated with the appropriate horseradisch peroxidase (HRP)-conjugated secondary antibody. The signals were routinely intensified employing enhanced chemoluminescence (ECL) or ECL-advanced detection kits (GE Healthcare, Buckinghamshire, UK).

### Statistical analysis

To evaluate significance of changes in protein amount Student's t-tests were applied, two sided p-values of p≤0.05 were taken as statistically significant. Cross-tabulations were calculated using Chi-quadrat test. For statistical analyses SPSS version 15.0 (SPSS Inc.) was used.

### Antibodies used for Western blotting

The following antibodies were applied: anti-ATF6 (1∶10,000; clone 70B1413.1; Imgenex, Hamburg, Germany), anti-β-actin (1∶10,000; clone AC-15; Sigma-Aldrich), anti-GRP78/Bip (1∶10,000; clone Gl-19; Sigma-Aldrich), anti-HIF1α (1∶500; clone 54; BD Transduction, Heidelberg, Germany), anti-vimentin (1∶4,000; clone H-84; Santa Cruz Biotechnology Inc., Heidelberg, Germany) and anti-ZEB1 (1∶500; HPA027524; Sigma-Life Science). For the quantification of ZEB1 its full length 170 kD form (arrow, Figure S1C) was taken (Swiss-Prot; Acc: P37275). For SDS-PAGE 25 µg protein were applied usually. For the detection of HIF1α, ZEB1 and ATF6 50 µg of protein were applied on gels. Specificity of the antibodies was checked by immunoblotting (Figure S1C).

### Immunofluorescence (IF)

SW480 and HCT116 cells were seeded on 12 mm round coverslides. After reaching appropriate densities, for EMT either as confluent or sparsely growing [19], and as confluent growing cultures for CoCl_2_ treatment (3 h, 100 µM CoCl_2_, serum free) [21], cells were fixed in the presence of 2% (v/v) formaldehyde. The following antibodies were employed: anti-β-catenin (1∶150; clone 14/Beta-Catenin, BD Biosciences, NJ, USA) and anti-E-cadherin (1∶100; clone EP6; Epitomics Burlingame, UK). Antibodies were diluted in 0.1% (w/v) saponin, counterstained with anti-Alexa-Fluor®488 (Invitrogen, Karlsruhe, Germany) and finally mounted in mounting medium containing DAPI (Vector Vectrashield Laboratories, Dossenheim, Germany). Immunofluorescence staining was detected by immunofluorescence-microscopy (Zeiss Axioscope 50, Zeiss Axiocam MRc).

### Patient Material

Formalin-fixed paraffin-embedded (FFPE) tissue from 16 CRC tumor cases was taken from the archives of the Department of Pathology, Ludwig-Maximillians Universität, München. Our study enrolled invasive colorectal adenocarcinomas pT2 and pT3 where single cells with mesenchymal morphology and nuclear β-catenin expression were found at the invasive front. The study complied with the requirements of the Ethics Committee of the Ludwig-Maximilian Universität of Munich.

### Immunohistochemistry (IHC)

Tissue sections (4 µm) of FFPE of human CRCs were prepared. For IHC the same antibodies as for Western blotting and IF were applied except for anti-HIF1α (clone EP1215Y; Epitomics, Burlingame, UK) which was used for IHC only. Endogenous peroxidase of sections was inhibited by 7.5% H_2_O_2_ at room temperature. Second antibody was used according to the ABC-Kit instructions. Slices were counterstaining for 10 sec. in Hematoxylin (Gill's Formula; Vector Laboratories), and coverslipped with Kaiser's glycerol gelatin (Merck) (Table S1).

## Results

### Induction of EMT in CRC cell lines by altering growth density

**Figure 1:**
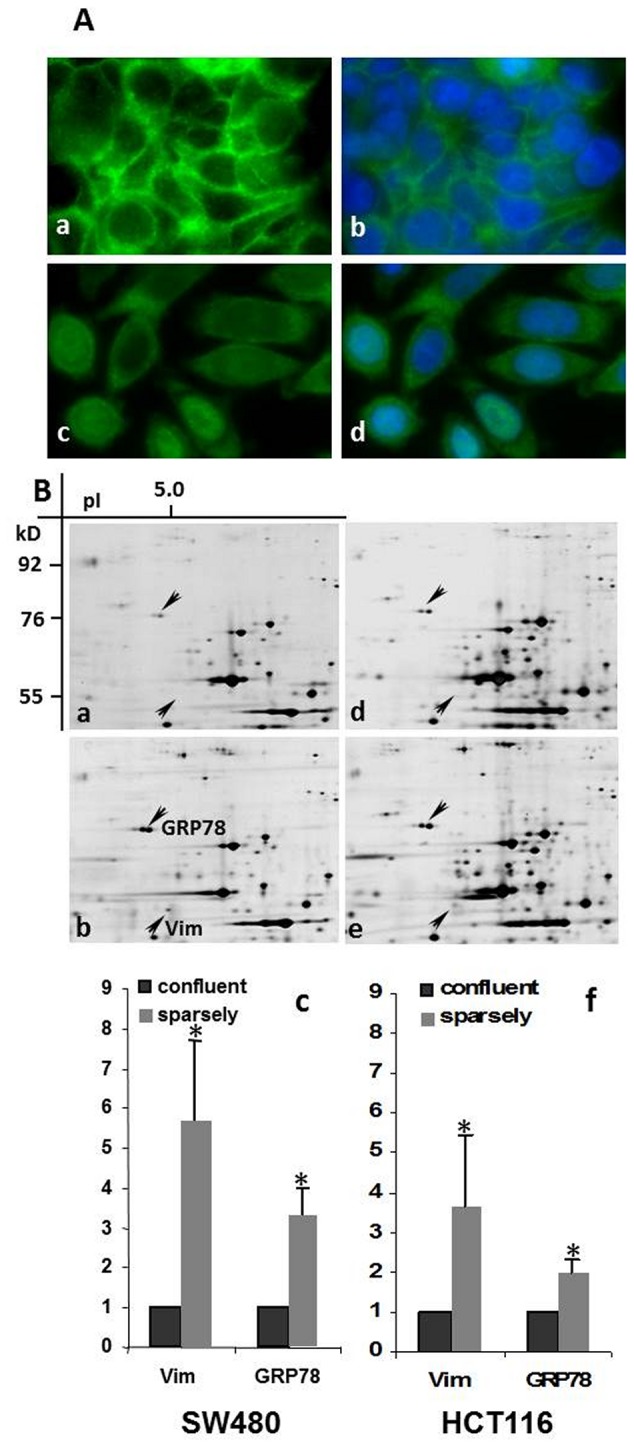
EMT is associated with an ER-stress response in CRC cell lines. **A**. Confluent growing SW480 cells are characterized by an epithelial growth pattern with membranous localization of β-catenin (a; green fluorescence). Sparsely growing cells, mimicking EMT display a mesenchymal growth pattern with cytoplasmic/nuclear localization of β-catenin (c; green fluorescence). Nuclei were counterstained by DAPI staining (b, d; blue fluorescence). (Magnification 630×). **B**. Quantitative changes in protein amount of the EMT-associated proteins vimentin (Vim) (located in 2-DE gels at 55.0 kD/pI 5.1) (arrows) and GRP78 (76.0 kD/pI 5.0) are presented in selected 2-DE areas: **a**. confluent SW480 cells; **b**. sparsely growing SW480 cells; **c**. quantification of the amount (n-fold) of vimentin (Vim) and GRP78 in confluent and sparsely growing SW480 cells **d**. confluent HCT116 cells; **e**. sparsely growing HCT116 cells. **f**. quantification of the amount (n-fold) of vimentin (Vim) and GRP78 in confluent and sparsely growing HCT116 cells. Data shown is the mean ± standard deviation (SD) from four independent experiments; * : p≤0.05.

First of all, we validated the experimental setup. For mimicking EMT *in vitro* SW480 and HCT116 cells were grown under different growth densities. The subcellular localization of β-catenin was taken as an indicator for epithelial- or mesenchymal organization using IF microscopy. Confluent growing tumor cells displayed an epithelial morphology characterized by the membranous localization of β-catenin (Figure 1A a, b). In contrast sparsely growing cells acquired a mesenchymal phenotype accompanied by a nuclear localization of β-catenin (Figure 1A c, d). Thus, the chosen experimental system allowed the growth of SW480 and HCT116 tumor cells resulting in an epithelial- or mesenchymal differentiation.

### Comparison of protein level in confluent and sparsely grown tumor cells

To investigate the effect of EMT on the constitution of the proteome, we compared 2-DE protein patterns of SW480 and HCT116 cells. The identity of at least thirty protein spots which showed a significant quantitative difference in intensity between confluent and sparse growth was determined by PMF. The significant increase in amount of the EMT indicator and mesenchymal marker protein vimentin [1], [2] in sparsely growing cells confirmed, that the chosen conditions in this experimental setting resulted in the differentiation between epithelial- and mesenchymal growth of the tumor cells (SW480: 5.8–fold, p = 0.0015, Figure 1B a, b, c; HCT116: 3.6-fold p = 0.01, Figure 1B d, e, f). Seven proteins were attributed as members of protein families of molecular chaperones. GRP78 attracted our attention due to its high protein amount found in sparsely growing cells, and its highly significant difference in the two growth patterns (SW480: 3.3-fold, p = 0.0005, Figure 1B a, b, c; HCT116: 2-fold, p = 0.0005, Figure 1B d, e, f; single analyzed 2-DE areas are shown in Figure S1B). As GRP78 is a well-known indicator of an ER-stress response [11], [13] its elevation indicated an interconnection between EMT and ER-stress response. Our view was supported by the finding of considerable amounts of the 50 kD-fragment of the stress sensor ATF6 in protein extracts of sparsely grown tumor cells (Figure S1D).

### Hypoxia induces both EMT and ER-stress in CRC cell lines

To validate the interrelationship of EMT and ER-stress we used the well-known stressor hypoxia. In lack of a hypoxic chamber we applied CoCl_2_ to simulate a hypoxia-like state in our cellular model [26], [27]. Therefore, confluent growing SW480- and HCT116 cells were exposed to CoCl_2_ for 1–9 h. Induction of a hypoxia-like state was found already 3 h after addition of CoCl_2_ by the appearance of HIF1α (Figure S2B, C).

IF-microscopy approved that under these conditions tumor cells displayed comparable molecular and morphological changes typical for EMT. SW480 cells lost cellular contacts and β-catenin and E-cadherin were dislocated from the membrane. β-catenin was mainly found in the cell nucleus and E-cadherin in the cytoplasm (Figure S2A a–f). HCT116 cells sustained cell contact, but β-catenin was no longer persistently but punctually arranged at cell margins. Moreover, the majority of β-catenin was now found in the cells' nuclei. E-cadherin was lost from the membrane and found in the cytoplasm (Figure S2A g–m). Additionally immunoblotting showed significant increased amounts of vimentin (H (hypoxia) 1 h: 1.2-fold, p = 0.095; H 3 h: 1.3-fold, p = 0.0003; H 6 h: 1.4-fold, p = 0.021; H 9 h: 1.3-fold, p = 0.0002 in SW480 cells and H 3 h: 1.22-fold, p = 0.116; H 6 h: 1.45-fold, p = 0.009 in HCT116 cells).

GRP78 and the 50 kD-fragment of the stress sensor ATF6 were also clearly elevated after 3 h in both cell lines using immunoblotting, thus indicating ER-stress (Figure S2B, C and D; GRP78: H 1 h: 1.04-fold, p = 0.42-fold; H 3 h: 1.18-fold, p = 0.019; H 6 h: 1.25-fold, p = 0.015; H 9 h: 1.29-fold, p = 0.02 in SW480 cells and H 3 h: 1.16-fold, p = 0.079; H 6 h: 1.3-fold, p = 0.004 in HCT116 cells).

Therefore the simulation of a hypoxia-like state applying CoCl_2_ caused both EMT and ER-stress in parallel.

### EMT is a prerequisite for induction of an ER-stress response in CRC cell lines

**Figure 2:**
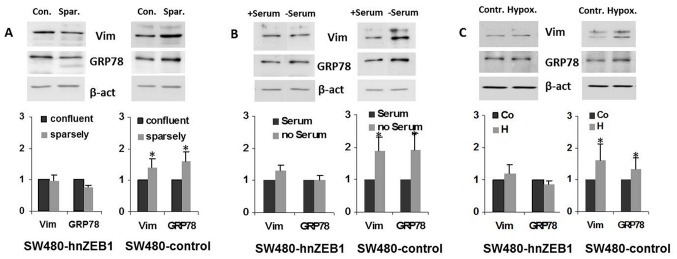
EMT is a prerequisite for ER-stress under conditions of sparse growth, serum starvation and hypoxia. **A**. SW480-shZEB1 clones show neither EMT indicated by low amounts of vimentin nor ER-stress indicated by low amounts of GRP78 under growth conditions that favor epithelial (confluent: con.) or mesenchymal (sparsely: spar.) growth conditions compared to SW480-control cells. **B**. SW480-shZEB1 clones do not develop EMT or ER-stress under conditions of stress induced by starvation (6 h- serum) compared to SW480-control cells. **C**. SW480-shZEB1 clones do not develop EMT or ER-stress under hypoxia-like conditions (6 h; serum free; 100 µM CoCl_2_) compared to SW480-control cells. Data shown is the mean ± SD from three independent experiments; * : p≤0.05.

Next, we wanted to investigate if there is a causative relationship between EMT and ER-stress response. Therefore we enrolled SW480-shZEB1 cells, which are characterized by their inability to undergo EMT [3]. Under conditions of sparse growth SW480-shZEB1 cells displayed significant lower signs of EMT indicated by vimentin but also less ER-stress indicated by GRP78 compared to SW480-control cells (SW480-shZEB1: vimentin: 0.9-fold, p>0.05; GRP78: 0.7-fold, p>0.05; SW480-control: vimentin:1.4-fold, p = 0.048; GRP78: 1.6-fold, p = 0.004; Figure 2A).

Next, we employed serum starvation as a second way to induce ER-stress [18]. Comparable results to conditions of sparse growth were found. In SW480-shZEB1 cells vimentin and GRP78 elevation was significant lower than in SW480-control cells (SW480-shZEB1: vimentin: 1.3-fold, p>0.05; GRP78: 1.02-fold, p>0.05; SW480-control: vimentin: 1.9-fold, p = 0.049; GRP78: 1.9-fold, p = 0.04; Figure 2B).

In a third approach SW480-shZEB1 and – SW480-control cells were grown to confluence and then incubated for 6 h in CoCl_2_ containing medium, as both EMT and ER-stress were pronounced after this time (Figure S2A, B and D). In this setting the amount of vimentin as well as GRP78 was not enhanced in SW480-shZEB-1 cells but considerably increased in SW480 controls similar to both preceding experiments (SW480-shZEB1: vimentin: 1.2-fold, p>0.05; GRP78: 0.8-fold, p>0.05; SW480-control: vimentin: 1.62-fold, p = 0.038; GRP78: 1.35-fold, p = 0.04; Figure 2C). Taken together, EMT turned out to be a prerequisite for the induction of an ER-stress response.

### Coupling of EMT and ER-stress response as well as hypoxia are also found in human CRCs

**Figure 3:**
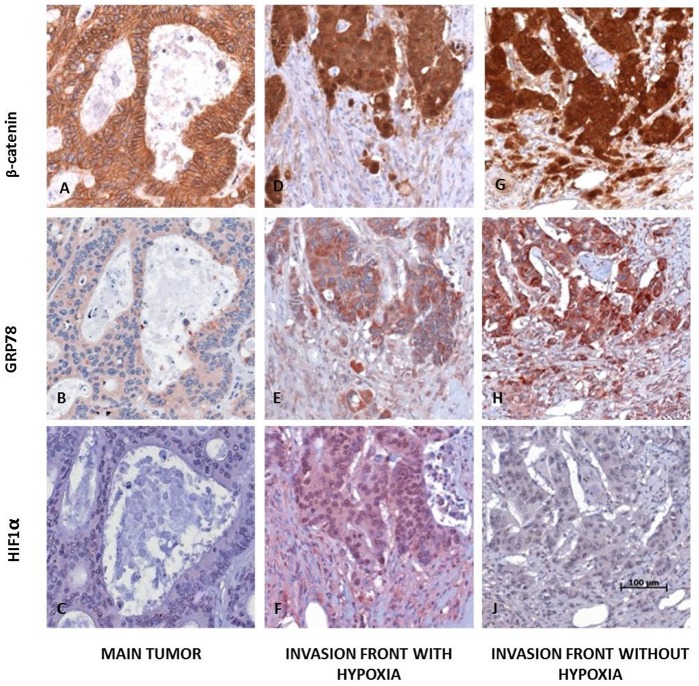
Human colorectal carcinomas with EMT show ER stress independent of HIF1α. In central tumor areas of human CRCs β-catenin was typically localized at the cell membrane (A) whereas only a weak staining was observed for cytoplasmic GRP78 (B) and HIF1α staining was found to be negative (C). At the invasion front strong nuclear β-catenin was detectable indicating EMT (D, G). In corresponding regions strong cytoplasmic GRP78 expression was found (E, H). In some of the cases an intense nuclear HIF1α staining was observed (F, with hypoxia), but not in others (I, without hypoxia) (magnification 200×; scale bar: 100 µm).

We wanted to validate if our findings were also found in human CRCs. Therefore serial sections of 16 CRCs with an elaborated invasion front containing mesenchymally differentiated cells were stained immunohistochemically with β-catenin, GRP78 and HIF1α. In central areas of tumors, where tumor cells displayed an epithelial phenotype, β-catenin was found mainly associated with the membrane (16/16 cases, 100%), while weak or absent staining was observed for GRP78 (14/16 cases, 88%) and HIF1α (16/16 cases, 100%) in all tumor cells (Figure 3 A–C). At the invasive front tumor cells displayed a strong nuclear expression of β-catenin (14/16 cases, 88%) which was found in 70–100% of tumor cells (Figure 3 D, G). GRP78 expression was markedly enhanced in 60–100% of tumor cells (14/16 cases, 88%) (Figure 3 E, H). In contrast, HIF1α staining was only found in some cases with an expression in 10–90% of tumor cells (6/16 cases, 38%; Figure 3 F) but not in others (10/16 cases 62%; Figure 3 I). Thus, the coupling of ER-stress with EMT which we found in our cellular *in vitro* system was also seen at the invasive front in CRCs (p = 0.046). Moreover we found a partial coupling of hypoxia and ER-stress which was not statistically significant (p = 0.433).

### ER-stress lasts longer than EMT in CRC cell lines after reoxygenation

**Figure 4:**
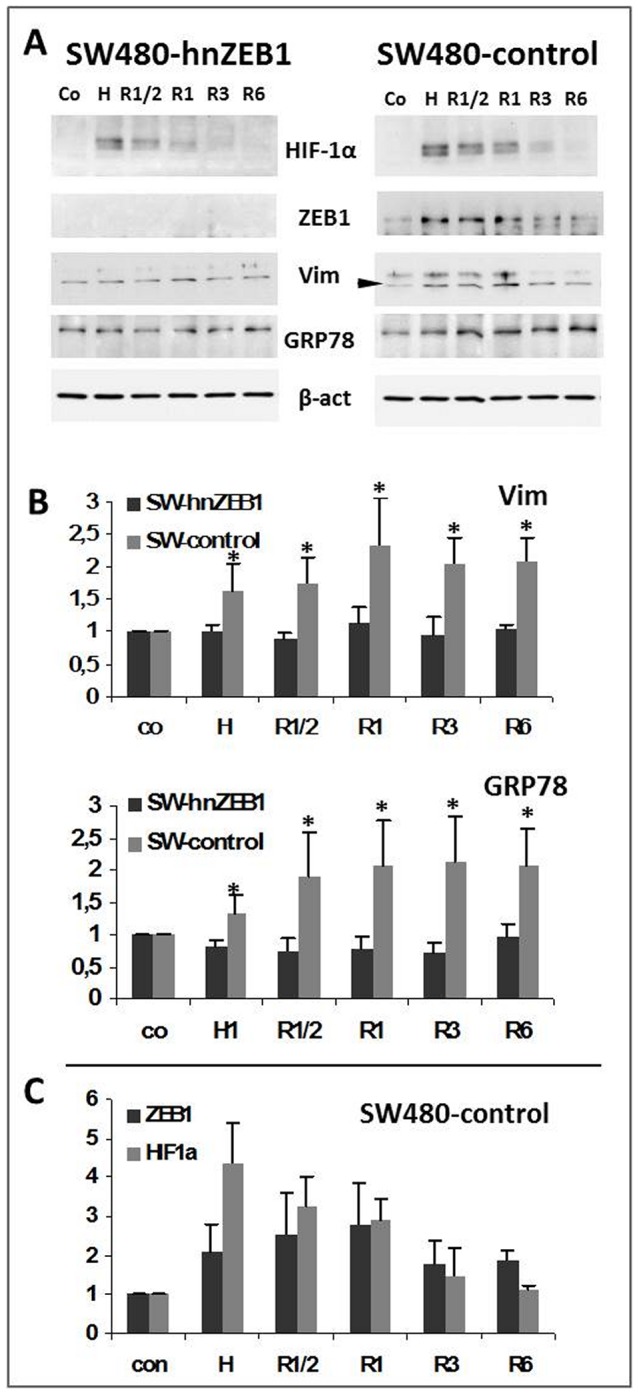
Development of EMT and ER-stress in SW480-shZEB1 and SW480-control cells under conditions of hypoxia and reoxygenation. **A** Confluent growing SW480-shZEB1 and SW480-control cells were exposed to normoxia and hypoxia-like conditions (6 h; serum free; 100 µM CoCl_2_) followed by reoxygenation (normal medium). Proteins were extracted at conditions of normoxia (control, Co), hypoxia (H) and reoxygenation (R) after ½h (R½), 1 h (R1), 3 h (R3) and 6 h (R6). Amounts of HIF1α, ZEB1, vimentin (Vim), GRP78 and β-actin (β-act as loading control) were determined. **B** Quantification of the amount (n-fold) of vimentin (Vim, arrow) and GRP78 in SW480-shZEB1 and SW480-control cells cultured under conditions of normoxia (control, Co), hypoxia (H) or reoxygenation (R) after ½h to 6 h (R½ - R6). **C**. The increasing/decreasing amounts of ZEB1- and HIF1α- after 6 h of exposition to CoCl_2_ (control, Co and hypoxia, H) followed by different reoxygenation-times (R½ - R6) are exemplarily shown for SW480-control cells. Data shown is the mean ± SD from three independent experiments; * : p≤0.05.

Finally, we wanted to find out if the partial coupling of hypoxia and ER-stress might be related to revascularization of tumors *in vivo*. Therefore in our *in vitro* system, the CoCl_2_ containing medium was replaced by normal culture medium thus mimicking reoxygenation. After 6 h of CoCl_2_-treatment tumor cells were incubated in normal medium for up to 24 h. After hypoxia, indicated by a considerable increase of HIF1α (Figure 4A, lanes: Co, H) reoxygenation resulted in the stepwise degradation of HIF1α in SW480-shZEB1 as well as SW480-control clones (Figure 4A, lanes: R½, R1, R3, R6, Fig. 4C).

Expectedly, in SW480-shZEB1 cells, no significant change regarding the amounts of vimentin and GRP78 was found during hypoxia (Figure 4A and B, lanes: Co, H) and reoxygenation (Figure 4A and B lanes; R½, R1, R3, R6; SW480-hnZEB1:vimentin: H 1.0-fold; R1/2: 0.9-fold; R1: 1.14-fold; R3: 0.95-fold; R6: 1.04-fold; all values: p≥0.05; GRP78: H: 0.8-fold; R1/2: 0.7-fold; R1: 0.8-fold; R3: 0.7-fold; R6: 0.96-fold; all values: p≥0.05).

In comparison in SW480-control clones, ZEB1 and vimentin increased initially (Figure 4A lanes R½, R1, Figure 4C) and were lost late during reoxygenation (Figure 4A lanes; R3, R6, Figure 4C; SW480-controls: vimentin: H: 1,62-fold; p = 0.029; R1/2: 1,73-fold, p = 0.006; R1: 2.3-fold, p = 0.02; R3: 2.06-fold, p = 0.002; R6: 2.07-fold, p = 0.01). Interestingly, the amount of GRP78 increased over reoxygenation-time in SW480-control cells (Figure 4A and B, lanes: R½, R1, R3, R6; GRP78: H: 1,34-fold; p = 0.011; R1/2: 1,89-fold, p = 0.02; R1: 2.06-fold, p = 0.017; R3: 2.12-fold, p = 0.011; R6: 2.1-fold, p = 0.014). Thus, during reoxygenation cells quickly loose the mesenchymal phenotype whereas ER-stress persists. Therefore ER-stress is associated with hypoxia but might also be found under normoxygenation.

## Discussion

### Correlation between EMT and ER-stress

In the progress of EMT epithelial cells change into dedifferentiated cells which display mesenchymal features. Activation of EMT promotes tumour cell dissociation and invasion which are prerequisite for metastasis to distant organs [1], [28]. Many CRCs are characterized by infiltrative growth at the invasive front which is associated with EMT. During malignant progression and infiltration, tumour cells are exposed to a variety of environmental conditions that induce structural alterations of secretory proteins leading to ER-stress and subsequently UPR [29]–[31]. In our experimental setting the mesenchymal phenotype, induced by changed growth conditions and hypoxia in CRC cell lines, was characterized by morphological changes, nuclear β-catenin translocation and significant increased amounts of the EMT indicator and mesenchymal marker protein vimentin. Additionally ER-stress response was indicated by elevation of the major player in ER-stress, the molecular chaperone GRP78, and the 50 kD-fragment of the stress sensor ATF6. Thus, our results suggest that EMT is correlated with ER-stress which is consistent with altered environmental requirements at the invasive front of CRCs.

### Hierarchy of cellular stress, EMT and ER-stress

**Figure 5:**
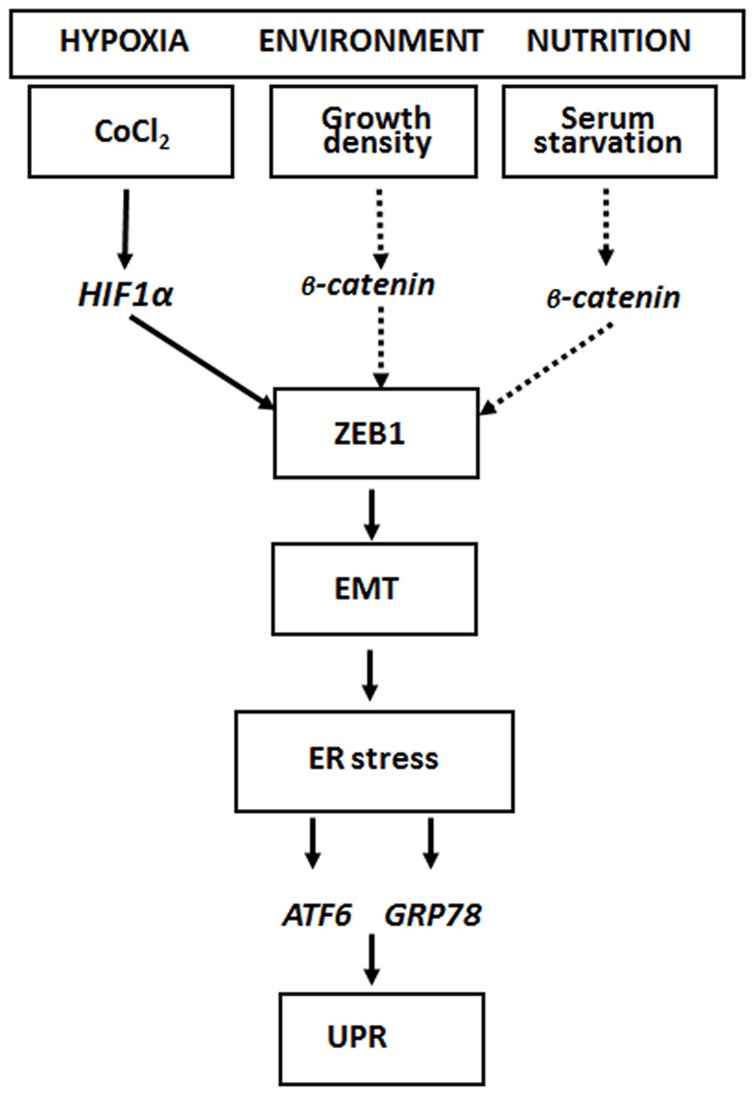
Model for the relationship between EMT and ER-stress. At the invasive front of CRCs cellular stress, like hypoxia or changes in the microenvironment, induces either the EMT regulator ZEB1 via HIF1 [1], [4] or potentially nuclear translocation of β-catenin [2], [4]. ZEB1 induces EMT which represents the driving force for ER-stress and UPR induction due to GRP78.

It is known that stress like hypoxia, changes in the microenvironment or nutrition might lead to the induction of EMT which is mediated by the action of the EMT master switch factor ZEB1 [3], [4]. In our experimental setting these stress conditions were all connected with an enhanced level of vimentin and with an ER-stress response characterized by high GRP78 amounts. In contrast, cells lacking ZEB1 displayed no EMT and no ER-stress response. EMT and ER-stress could be reverted by reoxygenation, which is also dependent on the presence of ZEB1. *In vivo*, at the invasive fronts of CRCs where tumour cells display strongly positive nuclear β-catenin and morphological signs of mesenchymal differentiation GRP78 intensity was also markedly enhanced. One mode of up-regulating the expression of ZEB1 is via the action of HIF1α the transcriptional mediator of hypoxia [1], [5], [32]. Another trigger for inducing ZEB1 is the transcriptional activity of β-catenin [33], [34] as it is found at the invasive front of CRCs [2], [4], [35]. According to our findings ER-stress depends essentially on the presence of ZEB1 and thus on EMT. Therefore cellular stress like hypoxia or malnutrition might activate HIF1α or β-catenin which induce ZEB1 and the global EMT program and finally lead to the induction of ER-stress. Our results based on hypoxia, serum starvation and growth pattern indicate that there is a hierarchy in the order: cellular stress→EMT→ER-stress (Figure 5).

### Elevation of molecular chaperones in CRCs

Molecular chaperones are involved in ER functions by proper protein folding and assembly as well as controlling the activation of transmembrane ER stress sensors [36]. Therefore, elevated levels of chaperones are observed in the context of ER-stress when an imbalance between the cellular demand for ER-function and ER-capacity occurs [11]. Increased expression of chaperones has also been reported in several types of cancer based on proteomic and immunohistochemical approaches [37]–[40]. In colon cancer elevated expression of chaperones was described as well as an incremental up-regulation in the adenoma-carcinoma sequence [41]–[43]. In support with these studies we found GRP78 a member of the chaperone family and well-known indicator of ER-stress response which showed a significant up-regulation in SW480 and HCT116 colon cancer cell lines under conditions of sparse growth. This situation corresponds with tumor cells at the invasive front in human CRCs showing EMT and was confirmed *in vivo* in our immuohistochemical approach (Figure 3).

### EMT and hypoxia as possible causes for ER-stress response

Hypoxia is a well-known activator of an ER-stress response. It occurs in most if not all solid tumors during their carcinogenesis [9], [10]. Moreover, it is a driving force for vascularization and progression of tumors. Furthermore, it is known, that diverse molecular and phenotypic changes induced by hypoxia are consistent with EMT [15], [16], [26]. In our system the simulation of hypoxia using CoCl_2_ caused ER-stress and induced EMT. But it remains an open question if the ER-stress response in the absence of hypoxia which was found at the invasive front of CRCs is due to EMT. Alternatively, it is conceivable that the invasive front is a zone with highly differing oxygen concentrations. Due to the different half-life times of HIF1α (∼30 min) [44] and GRP78 (∼72 h) [13] these zones with GRP78 but missing HIF1α expression might resemble areas of reoxygenation.

### Context specificity of ER-stress and EMT

In several disease patterns and tumor entities a different model of ER-stress and EMT has been proposed. In primary alveolar epithelial cells for example it has been suggested that ER stress induces EMT thereby contributing to lung fibrosis [45]. Similar models have been put forward for thyroid cell lines, chondrocytes and pancreatic beta cells [33], [34], [46], [47]. In contrast, in tubular epithelial cells albumin induced EMT and ER-stress have been described which are simultaneously activated through ROS and cSrc kinase [48]. Therefore the connection of EMT and ER-stress seems to be highly context specific. In our study we focused on CRCs with dedifferentiation of tumor cells at the invasive front and nuclear β-catenin accumulation due to an activation of the Wnt/β-catenin signaling. In this context our model of EMT induced ER-stress seems to take place at the invasive front and is supposed to be mediated by ZEB1. As colorectal carcinogenesis is very heterogeneous there might be even different connections between EMT and ER-stress in different types of colorectal tumors including microsatellite instable tumors (characterized by the absence of human MUT-L homologue 1 (hMLH1) expression) or tumors without dysregulated Wnt/β-catenin signalling [49].

### ER-stress correlates to stemness and might provide an alternative therapeutic approach

Interestingly, nuclear β-catenin is not only an inducer and indicator of EMT [7] but also of cancer stem cells (CSC) [50]. Moreover, induction of EMT results in the induction of tumor stemness [51]. Thus, the both cellular characteristics EMT and cancer stemness seem to be interdependent if not the same. Additionally, both traits, EMT and cancer stemness, are related to chemoresistance [52] and bad prognosis [53]. Including our results CSCs should then also be characterized *per se* by ER-stress. Therefore, the expression of GRP78 might be a surrogate marker of stemness [54] as well as chemoresistance [11], [55], [56]. Thus, our results are in support with the idea that targeting ER-stress might be an interesting additive element in a multimodal form of cancer therapy. This therapy would focus especially on CSCs in CRC which are known to be chemoresistant to the classical chemotherapeutic regimens [52]. Targeting the UPR pathways [57] as well as the expression of the GRP78 gene [58] are currently already in the focus of this research.

## Supporting Information

Figure S1 **Two dimensional gel, antibody testing and confirmation of 50 kD-ATF6 fragment.** **A. Two dimensional gel of cellular proteins from sparsely growing human HCT116 cells.** Evaluated area (framed): ∼47–96 kD/pI ∼4.2–6.1. **B. Single experiments.** Representative 2-DE areas from confluent (con.) and sparsely (spar.) grown SW480 and HCT116 cells are shown. Arrows: vimentin: Vim; GRP78. **C. Testing of antibodies used in this study:** Antibodies specific for β-actin (43 kD – mAb, lane 1); vimentin (55 kD - mAb, lane 2), GRP78 (75 kD – pAb, lane 3), ZEB1 (170 kD [arrow] –mAB, lane 4), together with β-actin (43 kD); HIF1α (120 kD – mAB, lane 5), ATF6 (50 kD fragment – mAb, lane 6; accompanied by an intense 25 kD band). The specificity of the used antibodies was determined by immunoblotting employing protein lysates made from SW480 or HCT116 cell lines. Specificity was granted when a single band or patterns of band were seen that represented the protein under investigation. **D. Abundance of the 50 kD-ATF6 fragment in SW480 cells.** Protein extracts of epithelially (confluent: con.) or mesenchymally (sparsely: spar.) growing SW480 cells as well as SW480 cells under hypoxica-like conditions (3 h; serum free; 100 µM CoCl2; control: co;) were separated by SDS-PAGE and immunoblotted.
(TIF)

Figure S2 **Hypoxia leads to EMT and ER-stress in CRC cells.** **A**. Hypoxia-like conditions alter localization of β-catenin and E-cadherin in confluent growing SW480 and HCT116 cells. Immunofluorescence-microscopy showed that confluent growing SW480 as well as HCT116 cells display an epithelial growth pattern with a membranous localization of β-catenin (a, g; green fluorescence and E-cadherin (b, h; red fluorescence, c, i: DAPI – blue fluorescence). After CoCl_2_ treatment (3 h, 100 µM CoCl_2_) SW480 cells changed to a more mesenchymal growing pattern associated with a cytoplasmic/nuclear localization of β-catenin and cytoplasmic localization of E-cadherin (d: green fluorescence, e: red fluorescence f: DAPI – blue fluorescence). HCT116 cells sustained cell contact, but β-catenin was observed predominantly in the nucleus, and E-cadherin in the cytoplasm, respectively (k: green fluorescence, l: red fluorescence, m:: DAPI – blue fluorescence). **B**. **C**. Confluent growing SW480 (B) and HCT116 (C) cells were cultured under conditions of normoxia or hypoxia-like conditions (serum free; 100 µM CoCl_2_, 1–9 h). Vimentin (Vim) was used as a mesenchymal marker and β-actin (β-act) as loading control. Enhanced amounts of GRP78 were verified after 3 h of CoCl_2_ addition. HIF1α was detectable after 3 h of CoCl_2_ incubation, the amount of the 50 kD-ATF6 fragment, was already enhanced after 1 h of addition of CoCl_2_. **D**. Quantification of the amount (n-fold) of vimentin (Vim) and GRP78 under normoxia and hypoxia-like conditions. Data shown is the mean ± SD from three independent experiments; * : p≤0.05.
(TIF)

Table S1 **Antibodies used for immunohistochemistry, dilution, incubation and detection systems.**
(DOCX)
