# Supplementary figures and images for: Epithelial-Mesenchymal Transition Induces Endoplasmic-Reticulum-Stress Response in Human Colorectal Tumor Cells
Source: PLoS One. 2014 Jan 31;9(1):e87386. doi: 10.1371/journal.pone.0087386 (PMC3909180; doi:10.1371/journal.pone.0087386)

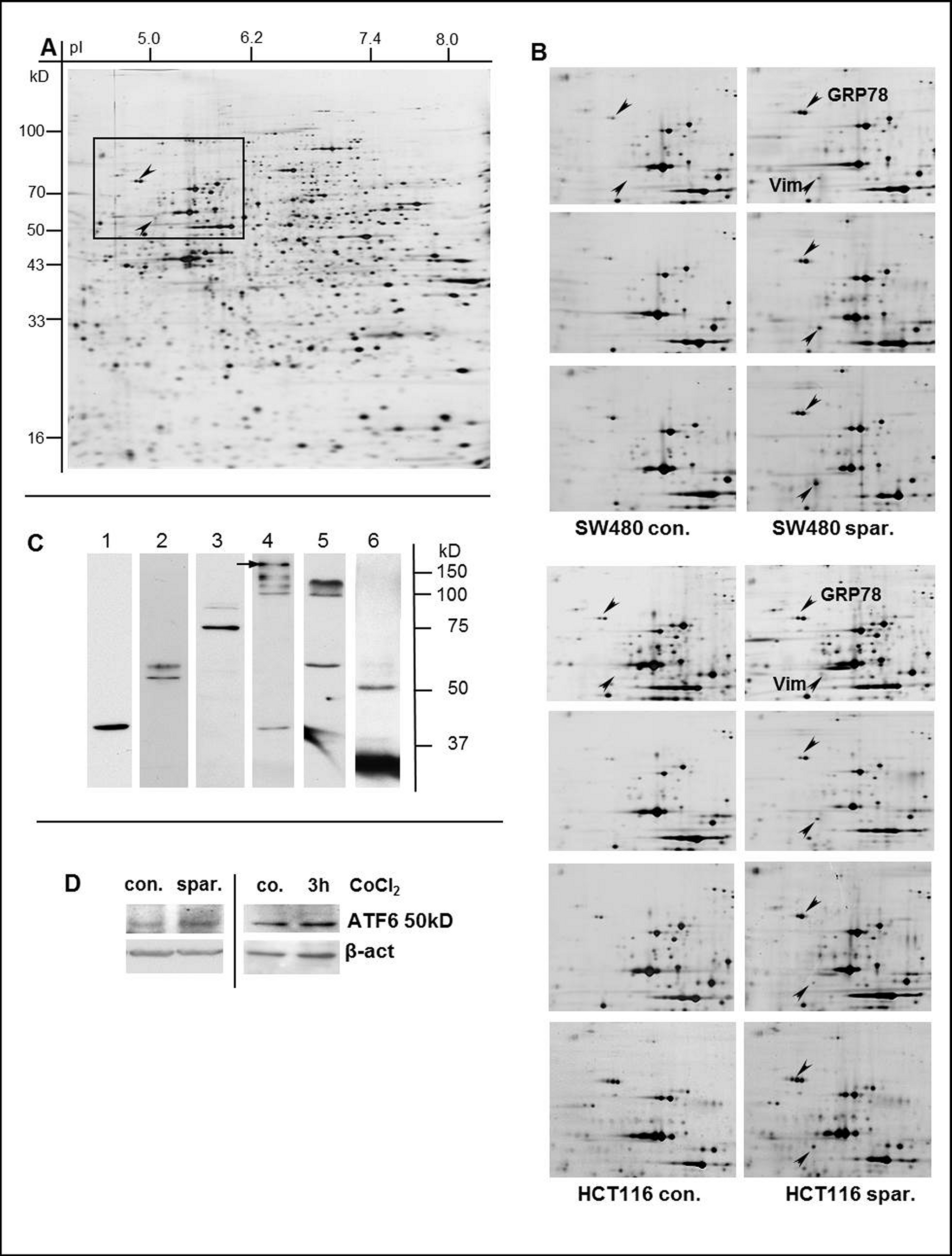

Supplement: Figure S1 — Two dimensional gel, antibody testing and confirmation of 50 kD-ATF6 fragment. A. Two dimensional gel of cellular proteins from sparsely growing human HCT116 cells. Evaluated area (framed): ∼47–96 kD/pI ∼4.2–6.1. B. Single experiments. Representative 2-DE areas from confluent (con.) and sparsely (spar.) grown SW480 and HCT116 cells are shown. Arrows: vimentin: Vim; GRP78. C. Testing of antibodies used in this study: Antibodies specific for β-actin (43 kD – mAb, lane 1); vimentin (55 kD - mAb, lane 2), GRP78 (75 kD – pAb, lane 3), ZEB1 (170 kD [arrow] –mAB, lane 4), together with β-actin (43 kD); HIF1α (120 kD – mAB, lane 5), ATF6 (50 kD fragment – mAb, lane 6; accompanied by an intense 25 kD band). The specificity of the used antibodies was determined by immunoblotting employing protein lysates made from SW480 or HCT116 cell lines. Specificity was granted when a single band or patterns of band were seen that represented the protein under investigation. D. Abundance of the 50 kD-ATF6 fragment in SW480 cells. Protein extracts of epithelially (confluent: con.) or mesenchymally (sparsely: spar.) growing SW480 cells as well as SW480 cells under hypoxica-like conditions (3 h; serum free; 100 µM CoCl2; control: co;) were separated by SDS-PAGE and immunoblotted. (TIF) [file pone.0087386.s001.tif]

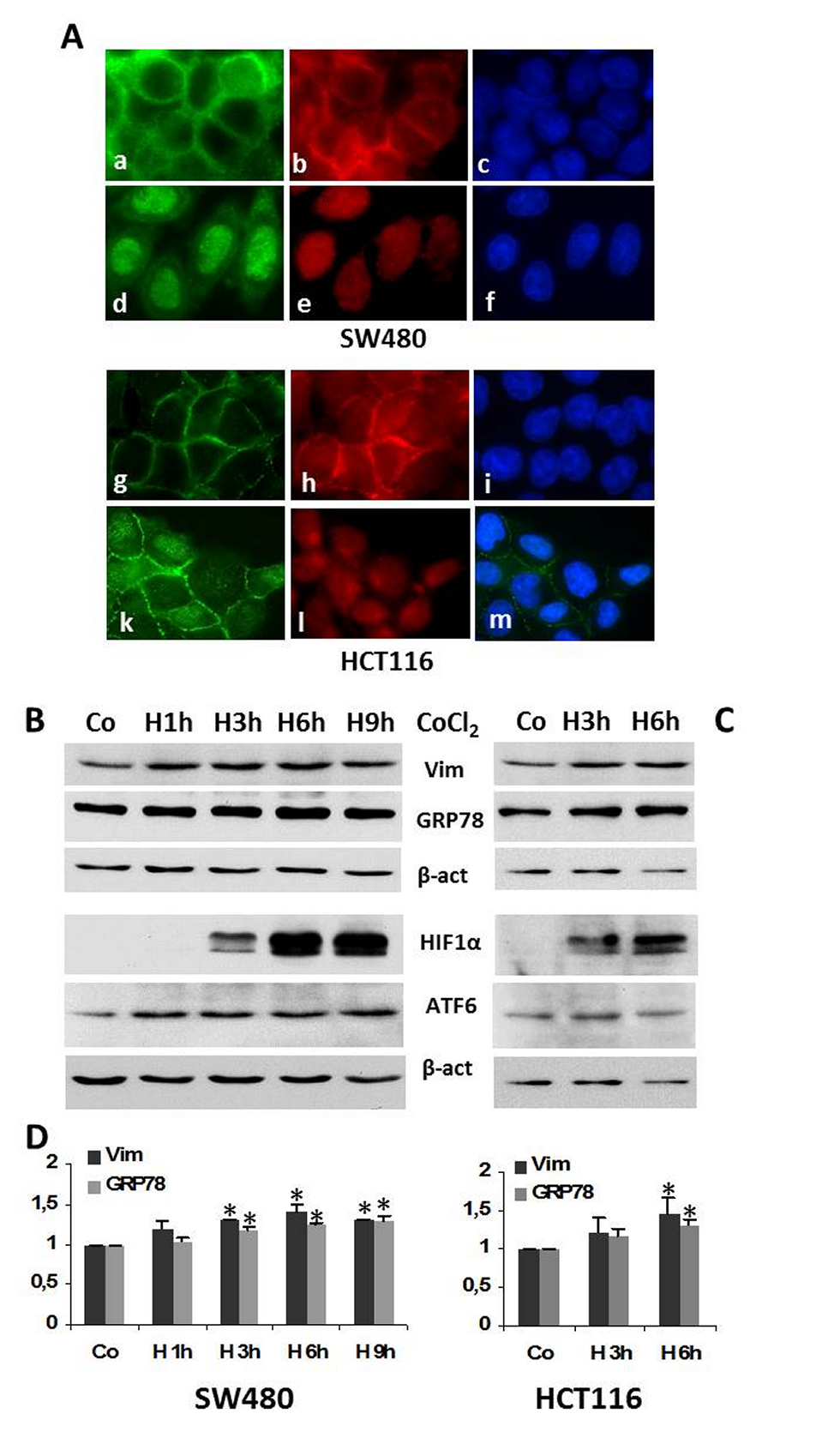

Supplement: Figure S2 — Hypoxia leads to EMT and ER-stress in CRC cells. A. Hypoxia-like conditions alter localization of β-catenin and E-cadherin in confluent growing SW480 and HCT116 cells. Immunofluorescence-microscopy showed that confluent growing SW480 as well as HCT116 cells display an epithelial growth pattern with a membranous localization of β-catenin (a, g; green fluorescence and E-cadherin (b, h; red fluorescence, c, i: DAPI – blue fluorescence). After CoCl2 treatment (3 h, 100 µM CoCl2) SW480 cells changed to a more mesenchymal growing pattern associated with a cytoplasmic/nuclear localization of β-catenin and cytoplasmic localization of E-cadherin (d: green fluorescence, e: red fluorescence f: DAPI – blue fluorescence). HCT116 cells sustained cell contact, but β-catenin was observed predominantly in the nucleus, and E-cadherin in the cytoplasm, respectively (k: green fluorescence, l: red fluorescence, m:: DAPI – blue fluorescence). B. C. Confluent growing SW480 (B) and HCT116 (C) cells were cultured under conditions of normoxia or hypoxia-like conditions (serum free; 100 µM CoCl2, 1–9 h). Vimentin (Vim) was used as a mesenchymal marker and β-actin (β-act) as loading control. Enhanced amounts of GRP78 were verified after 3 h of CoCl2 addition. HIF1α was detectable after 3 h of CoCl2 incubation, the amount of the 50 kD-ATF6 fragment, was already enhanced after 1 h of addition of CoCl2. D. Quantification of the amount (n-fold) of vimentin (Vim) and GRP78 under normoxia and hypoxia-like conditions. Data shown is the mean ± SD from three independent experiments; * : p≤0.05. (TIF) [file pone.0087386.s002.tif]
