# Supplementary material for: Epithelial-Mesenchymal Transition Induces Endoplasmic-Reticulum-Stress Response in Human Colorectal Tumor Cells
Source: PLoS One. 2014 Jan 31;9(1):e87386. doi: 10.1371/journal.pone.0087386 (PMC3909180; doi:10.1371/journal.pone.0087386)
Supplement: Table S1 — Antibodies used for immunohistochemistry, dilution, incubation and detection systems. (DOCX) [file pone.0087386.s003.docx]

| **Antibody incubation/dilution** | **Antigen retrieval system** | **Detection System** | **Chromogen** |
| --- | --- | --- | --- |
| **GRP78** (1h; 1:200) | **Histosafe-enhancer (pH:6)**  2x15min microwave:750W (Linaris GmbH, Wertheim-Bettingen, Germany) | **Vectastain Elite Universal ABC-kit** (Vector Laboratories, Burlingame) | **Aminoethyl carbazole (AEC)**  (Zymed Laboratories Inc.) |
| **HIF1α** (1h; 1:80) | **Epitope retrieval solution (pH:8)**  2x15min microwave:750W (Novocastra  Laboratories Inc., San Ramon) | **Elite Universal ABC-Kit**  (Vector Laboratories) | **AEC**  (Zymed) |
| **β-catenin**  (28min; 1:150) | **Epitope retrieval solution**  **CC1 (cell conditioning solution) (pH:8)**  36min at 95°C | **ultra View^TM^ Universal** | **DAB Detection Kit**  (all: Ventana Medical Systems Inc. according to the manufactures instructions) |
